# Integrated bioinformatics analysis for the identification of hub genes and signaling pathways related to circANRIL

**DOI:** 10.7717/peerj.13135

**Published:** 2022-04-25

**Authors:** Qiuyan Qin, Pengfei Zheng, Ronghui Tu, Jiegang Huang, Xiaoli Cao

**Affiliations:** 1 Department of Neurology, The First Affiliated Hospital of Guangxi Medical University, Nanning, Guangxi, China; 2 Department of Cardiology, The First Affiliated Hospital of Guangxi Medical University, Nanning, Guangxi, China; 3 Department of Geriatric Cardiology, The First Affiliated Hospital of Guangxi Medical University, Nanning, Guangxi, China; 4 The School of Public Health, Guangxi medical university, Nanning, Guangxi, China

**Keywords:** circANRIL, DEGs, Signaling pathways, Cell apoptosis, Bioinforamtics, Hub gene

## Abstract

**Background:**

Antisense noncoding RNA in the INK4 locus (ANRIL) is located on human chromosome 9p21, and modulation of ANRIL expression mediates susceptibility to some important human disease, including atherosclerosis (AS) and tumors, by affecting the cell cycle circANRIL and linear ANRIL are isoforms of ANRIL. However, it remains unclear whether these isoforms have distinct functions. In our research, we constructed a circANRIL overexpression plasmid, transfected it into HEK-293T cell line, and explored potential core genes and signaling pathways related to the important differential mechanisms between the circANRIL-overexpressing cell line and control cells through bioinformatics analysis.

**Methods:**

Stable circANRIL-overexpressing (circANRIL-OE) HEK-293T cells and control cells were generated by infection with the circANRIL-OE lentiviral vector or a negative control vector, and successful transfection was confirmed by conventional flurescence microscopy and quantitative real-time PCR (qRT-PCR). Next, differentially expressed genes (DEGs) between circANRIL-OE cells and control cells were detected. Subsequently, Gene Ontology (GO) biological process (BP) and Kyoto Encyclopedia of Genes and Genomes (KEGG) pathway enrichment analyses were performed to explore the principal functions of the significant DEGs. A protein–protein interaction (PPI) network and competing endogenous RNA (ceRNA) network were constructed in Cytoscape to determine circularRNA (circRNA)- microRNA(miRNA)-messenger RNA (mRNA) interactions and hub genes, and qRT-PCR was used to verify changes in the expression of these identified target genes.

**Results:**

The successful construction of circANRIL-OE cells was confirmed by plasmid sequencing, visualization with fluorescence microscopy and qRT-PCR. A total of 1745 DEGs between the circANRIL-OE group and control were identified, GO BP analysis showed that these genes were mostly related to RNA biosynthesis and processing, regulation of transcription and signal transduction. The KEGG pathway analysis showed that the up regulated DEGs were mainly enriched in the MAPK signaling pathway. Five associated target genes were identified in the ceRNA network and biological function analyses. The mRNA levels of these five genes and ANRIL were detected by qRT-PCR, but only COL5A2 and WDR3 showed significantly different expression in circANRIL-OE cells.

## Introduction

Antisense noncoding RNA in the INK4 locus (ANRIL) is located on human chromosome 9p21, within the p15/CDKN2B-p16/CDKN2A-p14/ARF gene cluster. Genome-wide association studies have identified single-nucleotide polymorphisms (SNPs) at 9p21 that are linked to susceptibility to coronary artery disease, ischemic stroke and tumors (Kong, Hsieh & Alonso, 2018; Chen et al., 2020; Congrains et al., 2012a; Sarkar et al., 2017). The causal variants at 9p21.3 regulate INK4/ARF expression and influence atherosclerotic vascular disease risk by modulating ANRIL expression, ANRIL can upregulate P14, P15 and P16 gene by recruiting polycomb repressive complex 1 (PRC1) and polycomb repressive complex 2 (PRC2), which are involved in vascular smooth muscle cell proliferation, plaque formation and arterial thrombosis to junctions (Holdt & Teupser, 2018; Holdt et al., 2016; Holdt et al., 2013). In an atherosclerosis (AS) rat model, reducing the expression level of circANRIL can prevent coronary atherosclerosis by decreasing apoptosis of vascular endothelial cells (VECs) and the expression of inflammatory factor, such as IL-1, IL-6, MMP-9, CRP and caspase-3, whereas the opposite effect was observed in circANRIL overexpressing (circANRIL-OE) groups, in which circANRIL promoted atherosclerotic plaque formation and atherothrombosis (Song et al., 2017). Earlier rat model experiments confirmed that inhibiting the expression of circANRIL in coronary heart disease can prevent atherosclerosis by reducing vascular endothelial injury, oxidative stress and inflammation (Shi et al., 2020). Another study confirmed that the expression levels of the linear and circular isoforms of ANRIL were not significantly correlated, indicating that the functions of circANRIL and linear ANRIL transcripts are independent of one another (Burd et al., 2010). Since circANRIL has a higher expression level than linear ANRIL and is more stable, it has received more attention from researchers.

Many researchers have demonstrated that circular RNA (circRNA) is a type of recently discovered endogenous noncoding RNA (ncRNA) that plays a vital role of posttranscription but has no ability to produce proteins (Prats et al., 2020). Some circRNA can regulate microRNA (miRNA)-mediated gene expression by serving as miRNA sponges. These circRNAs can interact with RNA-binding proteins, regulate protein translation and play an essential role in gene regulation (Zhou et al., 2020). However, the molecular function and biological role of circANRIL remain unknown (Hubberten et al., 2019; Holdt et al., 2016). It seems that circANRIL plays a critical role in cellular progression through different mechanisms. In this paper, an appropriate cell model was used to identify differentially expressed genes (DEGs) between circANRIL-OE HEK-293 cells and control cells and the possible involvement of these DEGs in the expression of particular genes and activity of signaling pathways. We intended to identify core genes and significant signaling pathways to further explore the potential mechanism by which circANRIL regulates cell proliferation and apoptosis. Our research will provide novel insight for exploring the mechanism of circANRIL in atherosclerosis and improving the therapeutic effect in atherosclerosis- related diseases.

## Materials and Methods

### Cell culture and construction of overexpression plasmid

The human cell line HEK-293T was purchased from the Cell Bank, Chinese Academy of Sciences (http://www.cellbank.org.cn) and the cells were cultured in high-glucose Dulbecco’s modified Eagle medium (DMEM) supplemented with 10% fetal bovine serum (FBS), 100 U/mL penicillin and 100 U/mL streptomycin, in a 37 °C, 5% CO_2_ and saturated humidity incubator. To generate the circANRIL- OE cell line, sequcences from the circRNA hsa_circ_0008574 were obtained from http://www.circbase.org/, we constructed an expression plasmid using the following sequence: CGGAATTC ***TGAAATATGCTATCTTACAG***TGTCCCTTTTGATGAGAAGAATAAGCCT CATTCTGATTCAACAGCAGAGATCAAAGAAAAGACTTCTGTTTTCTGGCCACCAGATAATGTTATCTGTGCTTAAAGAATTGAAAAACACACATCAAAGGAGAATTTTCTTGGAAA GAGAGGGTTCAAGCATCACTGTTAGGTGTGCTGGAATCCTTTCCCGAGTCA GCTTTCTAGAAGAAAACCGGGGAGATCTATTTGGAATGTATCTAACTCCAAAGAA ACCATCAGAGGTAACAGTAGAGACGGGGTTTCACCATGTTGGCCAGACTGGTCTT GAACTCCCGACCTCGTGATTCGCCCGCCTCGGCCTCCCAAAGTGCTGGGATTA CAGGTGTGAGACACCACGCCCGGCGGATAGAGAGAATTTTGACAG***GTGAATATA TTTTTTCTTGA***GGATCCCG. The restriction sites BamHI and EcoRI are underlined, and the splice sites were designed as donor sites (GT on the forward strand) and acceptor sites (AG on the reverse circularized sequence) which are shown in bold and italics, the other sites were target sequences. After digestion by the restriction enzymes EcoRI and BamHI, the PCR fragments were sequenced before cloning into the pUC57-Amp vector (Genewiz, suzhou, China) to generate the pUC57-hsa_circ_0008574 construct, which was transformed into Stbl3 competent cells (Invitrogen, Waltham, MA, USA). The status of individual colonies selected from plates was also confirmed by sequencing.

### Verification and analysis of the stable cell line

Lentivirus particles were generated by cotransfection of 4.4 µg pUC57-hsa_circ_0008574, 2.4 µg psPAX2 and 2 µg pMD2.G into HEK 293T cells with Lipofectamine™ 2000 (Invitrogen).The infection efficiency was determined under a fluorescence microscope after 48 h. Stably transfected cell lines were selected using puromycin (TaKaRa, Japan) and the cells were divided into three groups: circANRIL-OE group (transfected with the constructs), blank control group (untransfected cells) and negative control group (circRNA control, transfected with the empty vector). Each group contained three samples, and each sample contained approximately 1 × 10^6^ cells.

To validate the specificity of the HEK-293T cell transduction, total RNA was extracted from HEK-293T cells by using TRIzol reagent (TaKaRa, Japan) according the manufacturer’s protocol. mircoRNA (mRNA) levels of circANRIL and lincANRIL in these groups were analyzed with a qRT-PCR kit (TianGen, Beijing, China) following the manufacturer’s instruction.

### Identification of DEGs

According to the manufacturer’s protocol, a cDNA library was established by using the RNA Sequencing Library Preparation Kit (Illumina). The libraries were applied to a flowcell on a cBOT instrument (Illumina) to generate clusters, and then subjected to Illumina high-throughput RNA sequencing platform (Illumina, San Diego, CA, USA). To obtain clean data, the sequencing data were subjected to quality control, and the clean reads were mapped to the human reference genome (GRCh38) using TopHat (v2.1.0), The R function cor was utilized to obtain the Pearson correlation coefficient (Pearson’s r), the correlation coefficient ranged from 0 to 1, and higher scores indicate highly similar expression patterns among samples. Principal component analysis (PCA) was performed by R function prcomp, and the results were visualized using the ggfortify package version: 0.4.5 (https://cran.r-project.org/bin/windows/contrib/3.4/ggfortify_0.4.6.zip).

Differential expression analysis was performed using the edgeR language package version 3.4 (http://www.bioconductor.org/packages/release/bioc/html/edgeR.html) to screen for DEGs between the circANRIL-OE group and control groups. Raw counts were normalized with the TMM method, and then presented as log2 counts per million (LogCPM) values. We obtained the logFC and *P* value of each gene after assessing the data, and the adjusted *P* value (false discovery rate, FDR) was determined after multiple test correction with the Benjamini & Hochberg method, FDR < 0.05 and —logFC— > 1 were used as thresholds for DEG identification.

### Gene ontology (GO) and Kyoto encyclopedia of genes and genomes (KEGG) analyses of DEGs

All the DEGs were subjected to GO and KEGG pathway enrichment analyses. The analyses were performed using Database for Annotation, Visualization, and Integrated Discovery (DAVID, version 6.8, https://david-d.ncifcrf.gov/) functional annotation tools. GO biological process (BP) analysis was included in this study to explore the key signaling pathways. GO BP terms and KEGG pathway terms with a *P* value of < 0.05 and an enrichment score of >5 were defined as significantly enriched. Default DAVID database setting with medium stringency and Homo sapiens was defined as background.

### Protein–protein interaction (PPI) network

To construct the PPI network, the DEGs were input for PPI analysis into the Search Tool for the Retrieval of Interacting Genes/Proteins (STRING) database (version 10.0, http://www.string-db.org/). The species selected was “Homo sapiens”. The PPI score was set at 0.9 (highest confidence), Cytoscape (version 3.4.0, http://chianti.ucsd.edu/cytoscape-3.4.0/) was used to determine the PPI network pairs. The key topological properties of the PPI network were analyzed with the Cytoscape plug-in Network Analyzer (CytoNCA, version 2.1.6, http://apps.cytoscape.org/apps/cytonca) in Cytoscape (version 3.4.0). The parameter was set to ‘without weight’. All PPI pairs with a combined score >0.9 were extracted,Three parameters were chosen to evaluate every node in the network, including degree (the number of nodes directly linked to one node), betweenness (the relative importance of a vertex within the network) and closeness (the proximity of a network node to other nodes). the final combined scores were used to sequence and evaluate central proteins.

### Construction of the ceRNA network and hub gene selection

**Figure 1:**
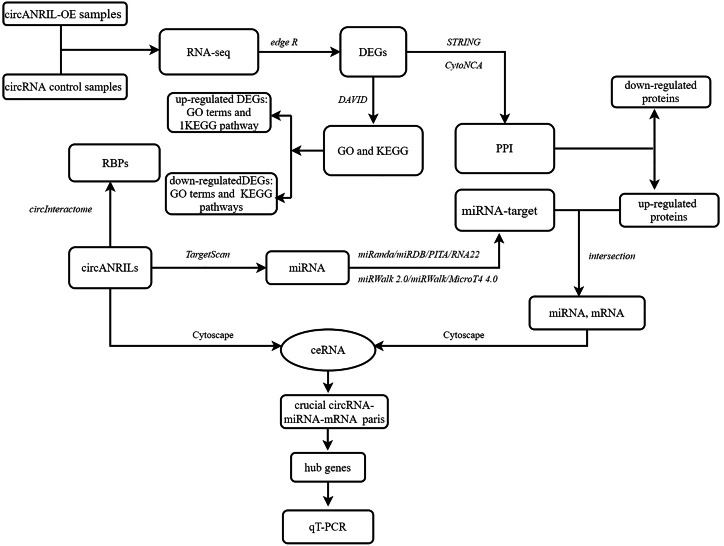
Flow diagram of study design. DEG, differentially expressed gene; GO, Gene Ontology; KEGG, Kyoto Encyclopedia of Genes and Genomes; PPI, Protein–protein interaction; RBP: RNA binding site protein.

To explore the underlying mechanism of circANRIL, the online database circInteractome (https://circinteractome.nia.nih.gov/) was used to identify RNA- binding proteins (RBPs) related to circANRIL and to determine whether any RBPs differentially expressed between the two groups. The TargetScan database (http://www.targetscan.org) was used to predict miRNAs within the 90th percentile. We then predicted the target genes of these miRNAs with miRWalk2.0 (http://mirwalk.umm.uni-heidelberg.de/), and the results of the following 6 databases were comprehensively considered to confirm the target genes: miRWalk (http://mirwalk.umm.uni-heidelberg.de/), MicroT4 4.0 (version 5; http://diana.imis.athena-innovation.gr), miRanda (https://anaconda.org/bioconda/miranda), miRDB (http://mirdb.org/miRDB/), PITA version 6 (https://web.archive.org/web/20200529050843/https://omictools.com/pita-tool) and RNA22 databases (https://cm.jefferson.edu/rna22/). Only those miRNA target genes included in all the gene sets were used for further analysis. Here, we reasoned that if circANRIL is upregulated, the same miRNA-regulated target genes should also be upregulated. Therefore, the potential miRNA-target gene pairs were obtained by overlapping the previously predicted target genes of the PPI and the upregulated DEGs. The circRNA-miRNA and miRNA-mRNA were combined for the construction of the circRNA-miRNA-mRNA regulatory network using Cytoscape 3.4.0. miRNAs and mRNAs with FDR <1%, absolute log2FC >2 and *P* < 0.01 were retained to establish the ceRNA network. Hub genes were selected according to the node degrees of the ceRNA network by calculating the number of circRNA-miRNA and miRNA-mRNA pairs. qRT-PCR was used for further validation of the selected genes. The overall procedure is summarized in the flow diagram in Fig. 1.

### Statistical analysis

Data in each group were assessed using SPSS 22.0 software (SPSS Inc.) and are presented as the mean ± SD. Differences between two groups were analyzed by the chi-square test and the Mann−Whitney U test. Comparisons between multiple groups were carried out using one-way analysis of variance or the Kruskal −Wallis H-test. *P* < 0.05 was considered statistically significant. Graphs were generated using GraphPad Prism 5 (GraphPad Software, San Diego, CA). All experiments were performed at least three times.

## Results

### Generation of a cell line stably overexpressing circANRIL

**Figure 2:**
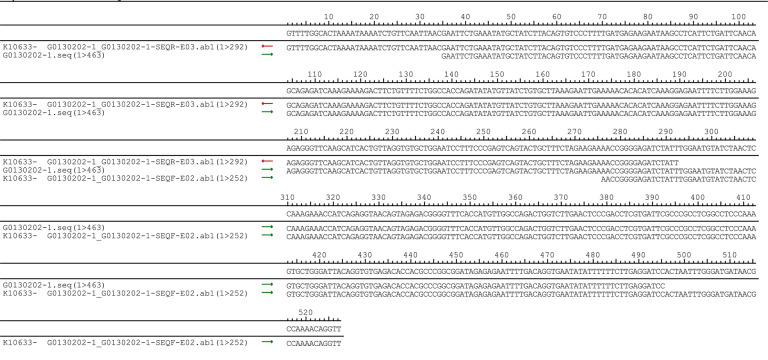
DNA sequencing confirmed of the pUC57-hsa_circ_0008574 recombinant.

**Figure 3:**
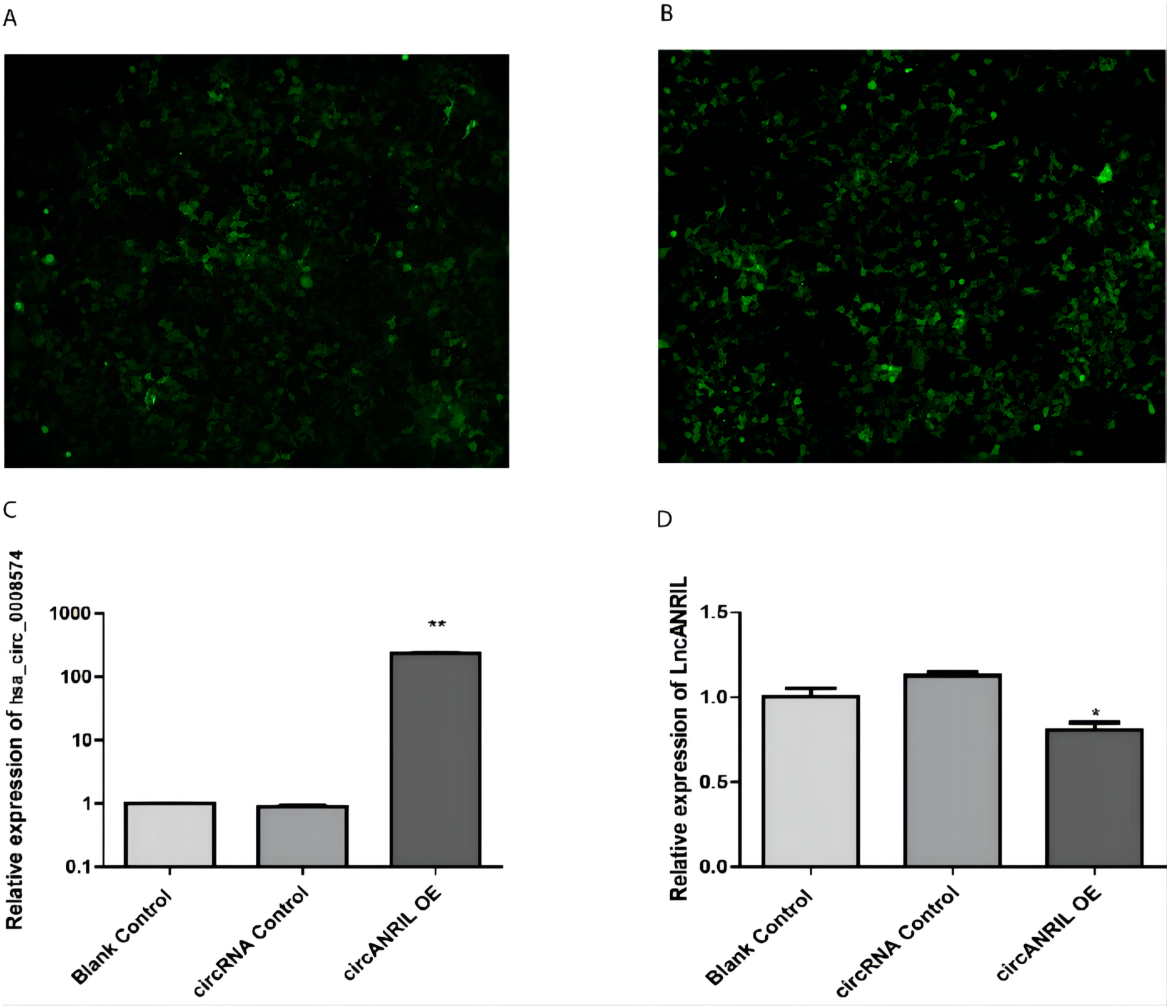
Transfection efficiency of each group of cells. (A) circRNA control. (B) circANRIL-OE group. (C) expression level of circANRIL in each group; (D) expression level of linear ANRIL in each group, ^∗^*P* < 0.05, ^∗∗^ *P* < 0.01 vs. blank control groups. Photographs were taken under an optical microscope with a magnification of 200×. Results were presented as mean ± SD. ^∗^*P* < 0.05, ^∗∗^*P* < 0.01, ^∗∗∗^*P* < 0.001. The Y axis is in a log scale in C. All of the experiments were performed in triplicate.

The pUC57-hsa_circ_0008574 recombinant expression vector was confirmed by sequencing analysis (Fig. 2). HEK-293T cells stably transfected with the pUC57-hsa_circ_0008574 recombinant plasmid were identified by green fluorescence (Figs. 3A, 3B). and qRT-PCR was used to determine the circANRIL overexpression efficiency in the corresponding cell line (denoted as circANRIL-OE). The circANRIL-OE group had a significantly higher circANRIL expression level than the negative and blank control group ( *P* < 0.05), while linear ANRIL showed a trend of decreased expression in the circANRIL-OE group compared to the control groups (Figs. 3C, 3D, Fig. S3-sheet 1).

### Functional analysis of the significant DEGs

**Figure 4:**
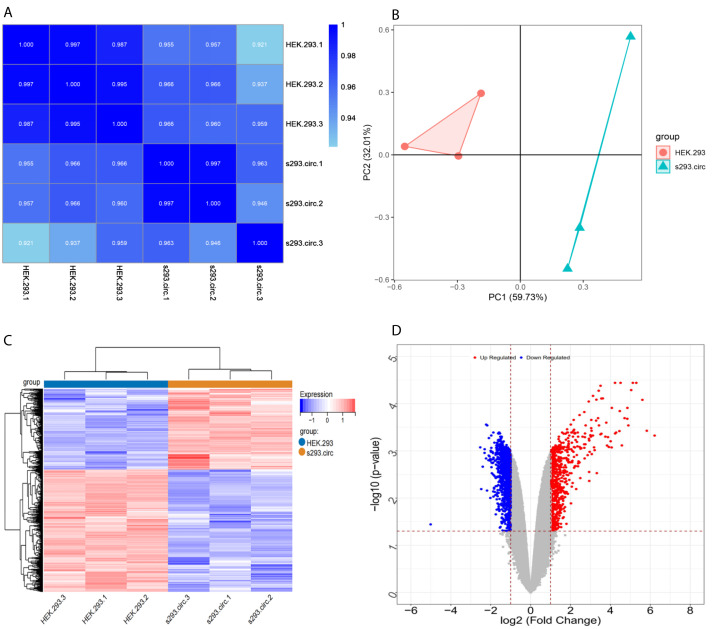
Screening for differentially expressed genes. (A) Correlation coefficient heat map of differential gene expression. (B) Principal Component Analysis (PCA) plots. (C) The differential genelist of circANRIL-OE significantly separates from control samples in hierarchical clustering. (D) Volcano plot for differentially expressed genes. Red color denotes up-regulation; blue represents down-regulation in circANRIL-OE group, respectively; gray indicates genes have no significant difference. *X* axis represents the *p*-value of the logarithmic transformation, and *Y* axis represents the average gene expression differences between circANRIL-OE samples and normal samples. |log2FC|>1 and *p*-value < 0.05 were set as the cut-off criteria.

Sequencing reads from an mRNA library constructed from total RNA from the two groups of cells were mapped to the human reference genome(GRCH38) by TopHat. GENCODE annotation of the human genes was performed by htseqcount (http://htseq.readthedocs.io/en/release_0.9.1/). After filtering out low quality reads, a total of 20,313 genes were further analyzed. All the raw sequencing data have been deposited into the SRA (accession number: PRJNA756043). The Pearson correlations(r) which ranged from 0.921 to 1, and *P* values are shown in (Fig. 4A). The median sample similarity between groups and within groups was 0.990 and 0.954, respectively, which indicated a significant difference between the circANRIL-OE and control groups. PCA analysis revealed significant separation between the two groups, two primary components (PC1 and PC2) that explained 91.74% of the total variance (PC1 = 59.73%, PC2 = 32.01%) (Fig. 4B). According to the threshold, in total, 1745 DEGs were identified, 1052 DEGs of which were upregulated and 693 of which were downregulated. The list of DEGs between the circANRIL-OE and control groups was subjected to hierarchical clustering. A volcano plot and a heat map of the DEGs are shown in Figs. 4C, 4D (Fig. S4-sheet 2).

### GO BP and KEGG analyses of the DEGs

**Figure 5:**
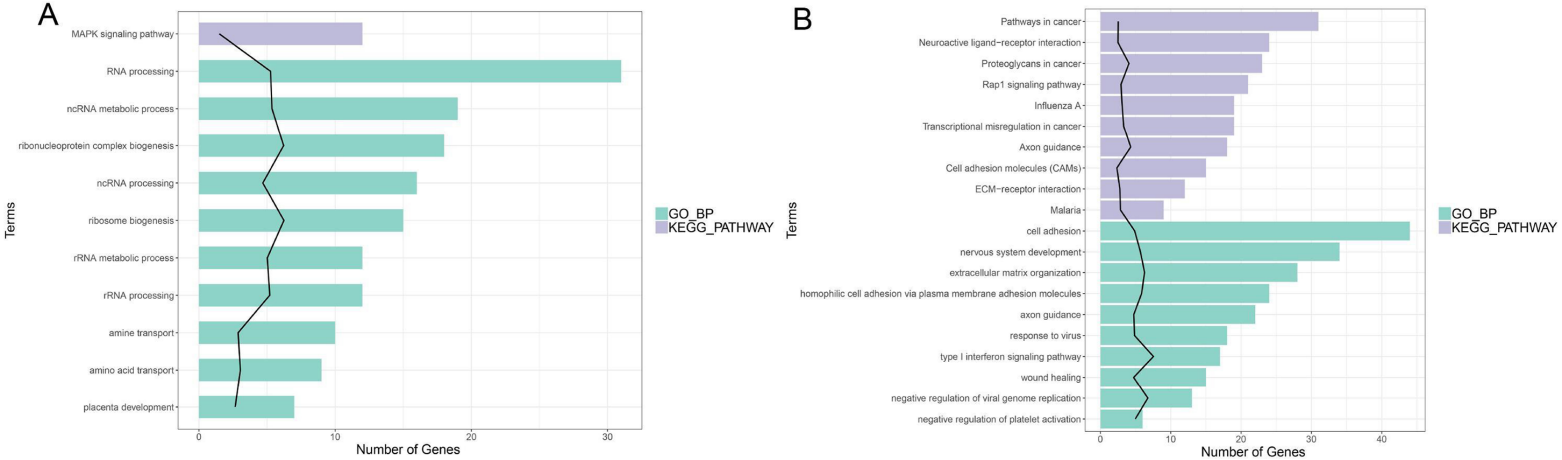
Function analysis of the significant DEGs between circANRIL-OE and control group. (A) The enriched top 10 GO biological processes (BP) and 1 KEGG pathways of the up-regulated significant DEGs. (B) The enriched top 10 GOBP and 10 KEGG pathways of the down-regulated significant DEGs.

In enrichment analysis, 26 GO BP terms were significantly enriched (*P* < 0.05) by the upregulated DEGs (*P* < 0.05) and 127 terms were enriched by the downregulated DEGs.The top 20 enriched GO BP terms are shown in Fig. 5. The results showed that the upregulated DEGs were significantly related to the following terms: RNA processing (GO: 0006396), regulation of cell proliferation (GO: 0042127), ncRNA metabolic process (GO: 0034660), ribonucleoprotein complex biogenesis (GO: 0022613), ncRNA processing (GO: 0034470), ribosome (GO: 0042254), RNA metabolic process (GO: 0016072), RNA processing (GO: 0006364), response to steroid hormone stimulus (GO: 0048545). The downregulated DEGs were predominantly associated with signal transduction (GO: 0007165), positive regulation of GTPase activity (GO: 0043547), cell adhesion (GO: 0007155), multicellular organism development(GO: 0007275), positive regulation of cell proliferation (GO: 0008284), nervous system development (GO: 0007399), negative regulation of cell proliferation (GO: 0008285), positive regulation of Apoptotic process (GO: 0043065), inflammatory response(GO: 0006954), and immune response(GO: 0006955). KEGG pathway analysis revealed that the upregulated DEGs were enriched in the MAPKsignaling pathway (hsa04010), while the downregulated genes were significantly enriched in terms 32 pathways in cancer (hsa05200), the PI3K-Akt signaling pathway (hsa04151), neuroactive ligand–receptor interaction (hsa0408), proteoglycans in cancer (hsa05205), HTLV-I infection (hsa05166), Rap1 signaling pathway (hsa04015), transcriptional misregulation in cancer (hsa05202), influenza A (hsa05164), cytokine-cytokine receptor interaction (hsa04060) and axon guidance (hsa04360) (Fig. 5; Fig. S5-sheet 3).

### PPI network analysis and hub gene selection

**Figure 6:**
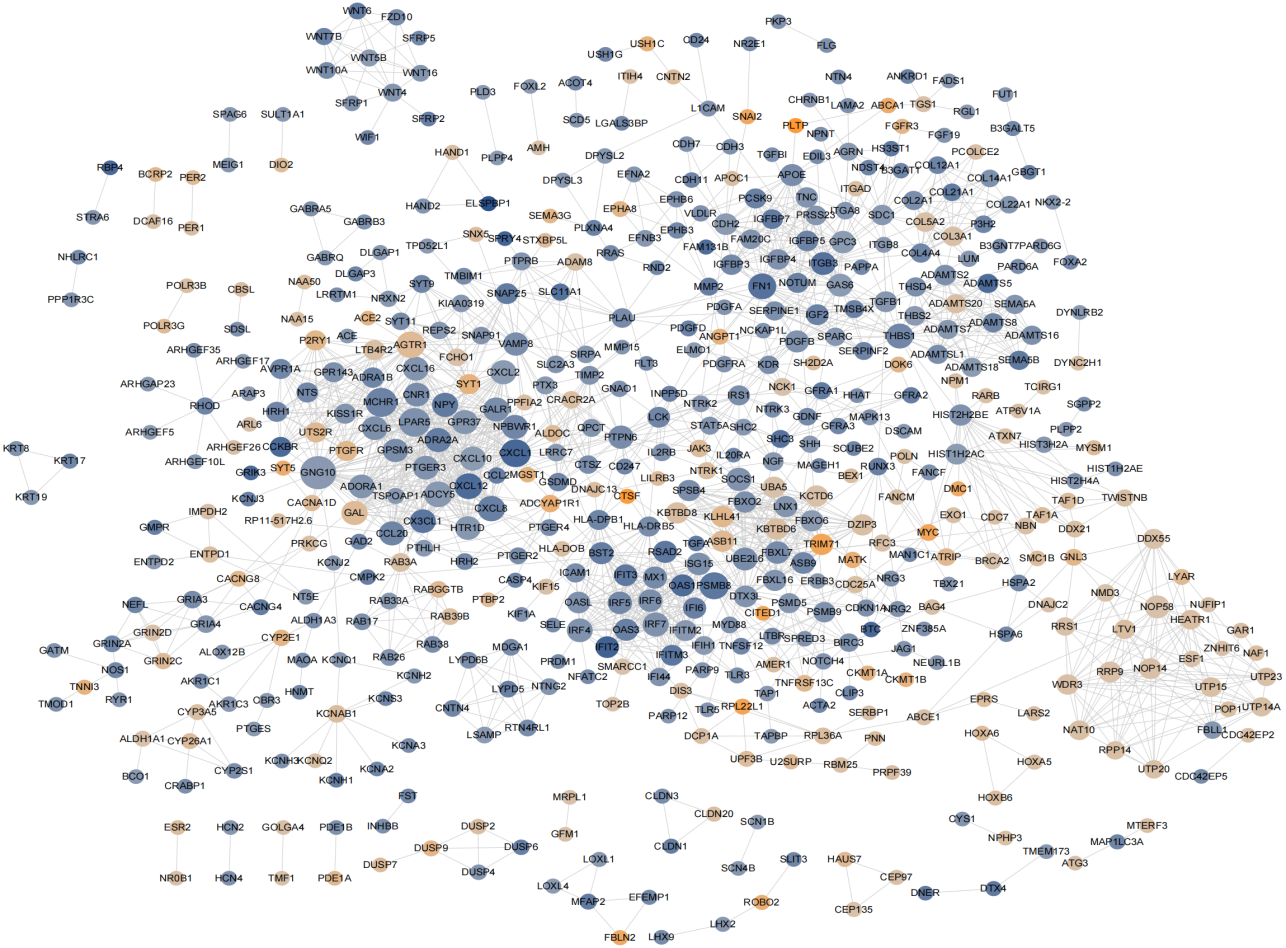
The hub genes determined in PPI networks. The intensity of the node color denotes the degree of upregulation (orange) or downregulation (blue), gray lines indicating protein-protein interactions, Node size reflects connectivity (the larger, the more connected).

According to previously described methods, a PPI network including 548 nodes,179 upregulated and 369 downregulated nodes was generated (Fig. 6; Fig. S6-sheet 4). The top 20 proteins ranked from the most to the least significant *P* values for degree, betweenness, and closeness are shown in Table 1. The results identified PSMB8, CXCL12, FN1, CXCL8, and THBS1 as potential core proteins in the PPI network because all three of their scores ranked in the top 20, However, these genes were excluded from further study because all of them were downregulated proteins.

**Table 1:** Topological properties of PPI network (top 20). The top 20 proteins ranked from the most to the least significant *P* values for degree, betweenness and closeness.

	Degree		Betweenness		Closeness
GNG10	46	THBS1	29293.018	FN1	0.008001986
MCHR1	36	LCK	23501.773	LCK	0.008000701
LPAR5	35	FN1	21341.697	ITGB3	0.007992882
CXCL1	33	GNG10	20834.193	PDGFB	0.007991134
PSMB8	31	VAMP8	20095.35	PTPN6	0.007990668
AGTR1	30	CXCL8	19591.28	IRS1	0.007990086
ADCY5	28	PLAU	19487.12	VAMP8	0.007989853
CXCL12	28	ITGB3	19444.19	PLAU	0.007989386
FN1	28	SNAP25	18873.1	STAT5A	0.007987756
CXCL8	27	PSMB8	16777.959	THBS1	0.007984032
THBS1	26	CXCL12	15816.917	CXCL12	0.007983218
GPR37	24	HIST2H2BE	15608.454	SERPINE1	0.007981357
NPBWR1	24	HIST1H2AC	13979.287	IGF2	0.007980079
ADRA2A	24	TWISTNB	13411.671	CXCL8	0.007979614
CXCL2	24	IRS1	13227.699	TGFB1	0.007979614
CXCL10	24	PTPN6	12547.569	KDR	0.007977988
NPY	24	GAD2	12330	SIRPA	0.007977756
CCL20	24	DIS3	11803.323	HLA-DRB5	0.007977291
IRF7	24	AGTR1	11732.014	HLA-DPB1	0.007977291
GAL	24	ALDH1A3	11584	PSMB8	0.007977059

The RBPs of 13 circRNAs were identified via circBase (http://www.circbase.org/) (Table 2). The search yielded six flanking region binding sites (corresponding to EIF4A3, AUF1, U2AF65, FUS, TIAL1, and PTB), but the corresponding genes were not differentially expressed between the groups.

**Table 2:** The 13 circRNAs derived from ANRIL and predicted RBPs. circANRIL and their predicted RBPs from circBase.

Position	Strand	circRNA ID	Genomic length	Spliced length	Gene symbol	RBPs
chr9:22056250-22064017	+	hsa_circ_0086590	7767	981	CDKN2B-AS	EIF4A3
chr9:22046749-22097363	+	hsa_circ_0008796	50614	1719	CDKN2B-AS	EIF4A3, U2AF65
chr9:22046315-22097363	+	hsa_circ_0004297	51048	1852	CDKN2B-AS	EIF4A3
chr9:21994789-22066352	+	hsa_circ_0086587	71563	2448	CDKN2B-AS	EIF4A3, FUS, PTB
chr9:22046749-22112394	+	hsa_circ_0004735	65645	1795	CDKN2B-AS	AUF1, EIF4A3, U2AF65, TIAL1
chr9:22046749-22065756	+	hsa_circ_0086589	19007	1350	CDKN2B-AS	EIF4A3, U2AF65, FUS
chr9:22046749-22056386	+	hsa_circ_0008574	9637	409	CDKN2B-AS	EIF4A3, U2AF65, PTB
chr9:22046315-22065756	+	hsa_circ_0086588	19441	1483	CDKN2B-AS	EIF4A3, FUS
chr9:22056250-22065756	+	hsa_circ_0086591	9506	1077	CDKN2B-AS	EIF4A3, FUS
chr9:22043037-22097363	+	hsa_circ_0003306	54326	5130	CDKN2B-AS	EIF4A3, PTB
chr9:22046749-22064017	+	hsa_circ_0005118	17268	1254	CDKN2B-AS	U2AF65, EIF4A3
chr9:22058357-22066352	+	hsa_circ_0086592	7995	1060	CDKN2B-AS	EIF4A3, FUS, PTB
chr9:22046749-22066352	+	hsa_circ_0004226	19603	1469	CDKN2B-AS	EIF4A3, FUS, PTB, U2AF65

**Figure 7:**
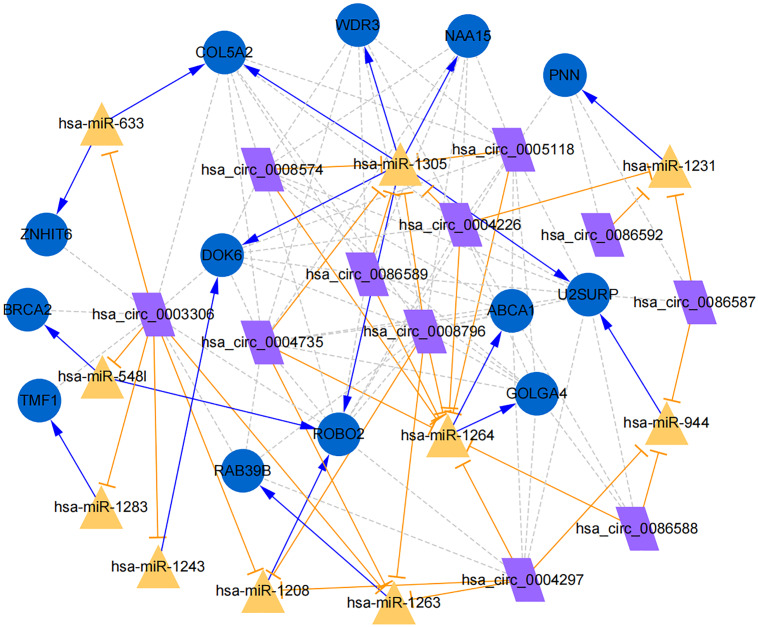
Establishment of circRNA-miRNA-gene network based on Cytoscape software. Regulation of hub genes in circRNA-miRNA-gene network. Orange triangle nodes stand for DEmiRNA, blue circular nodes stand for the target genes and purple rhombus nodes represent circRNAs from ANRIL. The lines represent the regulation of relationship between two nodes.

**Figure 8:**
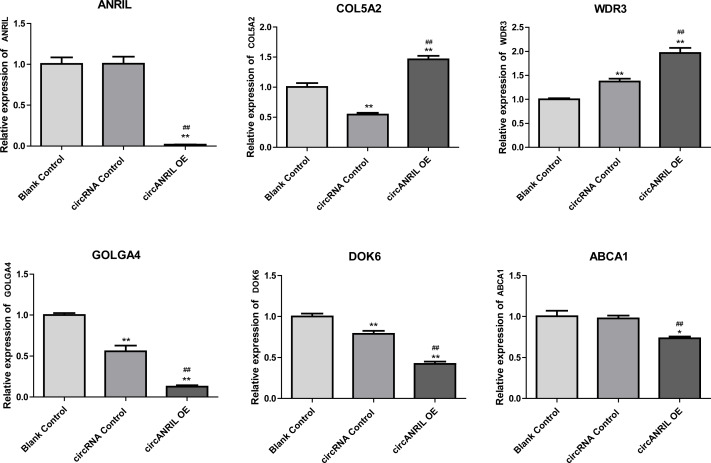
mRNA expression levels of six hub genes verified by qRT-PCR analysis. ^∗^*P* < 0.05, ^∗∗^*P* < 0.01 *vs.* blank control.

Moreover, the miRNA targets of the 13 circRNAs of ANRIL were predicted using the TargetScan database (http://www.targetscan.org). A total of 197 circRNA-miRNA pairs including 13 circRNAs and 56 miRNAs were obtained, and 2456 miRNA-targets of these 56 miRNAs were predicted by miRWalk2.0 and six other databases. Based on the previously mentioned methods, the overlapping genes of these predicted mRNAs and genes encoding the upregulated proteins of the PPI network were determined for 36 miRNA-target pairs, including 22 miRNAs and 20 target genes. Furthermore, 71 circRNA-miRNA-mRNA pairs were obtained by identifying miRNAs and target genes regulated by the same miRNAs through intergration of the circRNA-miRNA and miRNA-mRNA relationships. The circRNA-miRNA-mRNA network contained 31 circRNA-miRNA pairs, 69 miRNA-mRNA coexpression relationships, and 18 miRNA-mRNA pairs. There were a total of 34 nodes, including 11 circRNAs, 10 miRNAs and 13 mRNAs. Among them, miR-1305 had the most miRNA target sites (six) while miR-1264 had two miRNA target sites, indicating that miR-1305 and miR-1264 serve as crucial miRNAs regulating the ceRNA network (Fig. 7; Fig. S7-sheet 5, sheet 6, sheet 7). There were a total of eight target genes regulated by these two miRNAs: COL5A2 (ID: 1290), DOK6 (ID: 220164), ABCA1 (ID: 19), GOLGA4 (ID: 2803), WDR3 (ID: 10885), NAA15 (ID: 80155), ROBO2 (ID:6092) and U2SURP(ID:23350). Based on their biological functions, five of these genes are possibly related to circANRIL regulation in the ceRNA network were assessed with qRT-PCR analysis:COL5A, DOK6 , ABCA1, GOLGA4 , and WDR3. ANRIL(ID: 100048912) is known to be related to altered circANRIL expression, and was thus also included in the qRT-PCR validation (Table 3). As shown in Fig. 8 (Fig. S8-sheet 8), only COL5A2 and WDR3 mRNA expression was markedly increased in the circANRIL-OE cell line, while the others showed decreased expression.

## Discussion

Recent studies on circRNA-miRNA interactions have indicated that circRNAs play a critical role in the regulation of gene expression by interacting with their target miRNAs. A study found that circANRIL expression is related to INK4/ARF transcription and atherosclerotic disease susceptibility (Burd et al., 2010). The genotype of chromosome 9p21 affect the balance between linear ANRIL and circANRIL levels in vascular smooth muscle cells (VSMCs) and macrophages. Changes in the level of circANRIL regulate cell apoptosis and inflammation responses (Song et al., 2017; Muniz et al., 2021; Holdt et al., 2016; Congrains et al., 2012b). Therefore, the effect of high levels of circANRIL, which inhibits cell proliferation and apoptosis in the early stage of atherosclerotic plaque development in human vascular tissues on atherosclerosis is controversial. In the present study, we constructed a circANRIL-OE cell line by transfecting the pUC57-hsa_circ_0008574 vector into HEK-293T cells. qRT-PCR analysis results showed that the circANRIL expression level was significantly upregulated in the circANRIL- OE group compared with the control groups, while the linear ANRIL expression level was mildly decreased, This result indicated successful construction of circANRIL- OE cell line.

**Table 3:** Primer sequences of the target genes forqRT-PCR.

Gene	Gene description	Primer sequences	
		Forward	Reverse
ANRIL	antisense non-coding RNA in the INK4 locus	TCCAGTGCAAGTATGGTCTGTGATC	GCAAAGCATAAATCTTGACTCTGGC
COL5A2	Collagen Type V Alpha 2 Chain	GACTGTGCCGACCCTGTAAC	CCTGGACGACCACGTATGC
DOK6	Docking protein 6	TCGTGAAAGATGATTGCCACC	TCGTGAAAGATGATTGCCACC
ABCA1	ATP-binding cassette A1	ACATCCTGAAGCCAATCCTGA	CTCCTGTCGCATGTCACTCC
GOLGA4	golgin A4	CTCTGCAAGAACAACTGGATGA	TCACGCAACTGAGTGATAAGTTT
WDR3	WD repeat domain 3	GGCAGTACCAGCTTGTGAACA	GGGGCATAAGCAAGTAACTTCT
GAPDH	Glyceraldehyde-3-Phosphate Dehydrogenase	TGACAACTTTGGTATCGTGGAAGG	AGGCAGGGATGATGTTCTGGAGAG

To explore the potential mechanisms of circANRIL regulation, we firstly compared the expression of RBPs binding to circANRIL in these two groups, but no significant findings were revealed which indicated that circANRIL overexpression is independent to RBPs binding states. Furthermore, various bioinformatics analysis methods were performed to reveal the core genes and key pathways related to circANRIL function. According to the GO BP results, the DEGs were mainly related to the following cellular processes: RNA biosynthesis and processing, cell proliferation and signal transduction. As reported in previous studies, circANRIL is involved in the maturation of VSMCs and macrophage rRNAs. When circANRIL binds to PES1, pre-rRNA treatment and ribosome biosynthesis are impaired, resulting in nuclear pressure, activation of p53, and a subsequent increase in cell apoptosis and reduction in the proliferation rate, This finding of the GO BP analysis is in line with the results of previous study.

In KEGG pathways analysis, the downregulated DEGs were enriched in 32 significant KEGG pathways, but only the MAPK signaling pathway was identified for the up-regulated DEGs, Twelve of the upregulated DEGs subjected to the pathway had gene IDs suggesting that they were related to the MAPK signal pathway, MAPK belongs to the serine/threonine protein kinase family and plays a critical role in transporting signal from the cytoplasm to the nucleus and causing nuclear changes. In addition, MAPK families have been proven to be essential for controlling diverse cellular behaviors, including cell proliferation, differentiation and apoptosis. For instance, the p38 MAPK pathway, ERK1/2 MAPK pathway and JNK signaling pathway. P38 MAPK and JNK pathways can be activated by inflammation, ischemia/reperfusion, oxidative stress, and other injuries related to atherosclerosis and vascular disease (Sui et al., 2014; Xiong et al., 2015). Moreover, Phosphorylated p38 MAPK can activate the NF-kB signaling pathway to enforce the inflammatory response, Abnormalities in ANRIL expression in VECs involve inflammation and accelerate endothelial injury through the TNF- *α*-NF-k β-ANRIL/YY1-IL6/8 signaling pathway (Holdt et al., 2016). Low expression of circANRIL induced cell apoptosis and the inflammatory response in OGD/R-induced HBMECs by sponging miR-622, a process that inhibited the NF-k β pathway (Jiang et al., 2020). However, the precise role of P38 MAPK in circANRIL function is still unknown. The downregulated DEGs were enriched in 32 significant KEGG pathways, and the pathways most enriched by these genes were tumor-related pathways, since the cell cycle and development of tumors are closely related to intensive proliferation and inhibited apoptosis. Overexpression of ANRIL has been found in several malignant tumors, and abnormally high expression of ANRIL was found to be related to tumor proliferative ability (Kong, Hsieh & Alonso, 2018), but there was no association between circANRIL and these pathways in this study.

In the analysis of the PPI network and ceRNA network, five downregulated proteins (PSMB8, CXCL12, FN1, CXCL8 and THBS1) were identified as core protein in the PPI network, but their change trend differ from that predicted for circANRIL. In the ceRNA network, five hub genes with a high degree of interaction, COL5A2, DOK6, ABCA1, GOLGA4 and WDR3 were identified as hub genes of circANRIL, which was further confirmed by qRT-PCR validation, The results demonstrated that COL5A2 and WDR3 mRNA expression was markedly increased in the circANRIL- OE cell line compared to the control cell lines, as predicted. while ANRIL, ABCA1, DOK6 and GOLG4 mRNA expression was remarkably decreased. COL5A2 is a subtype of collagen V involved in the pathogenesis of fibrosis as a regulatory fibril-forming collagen. The COL5A2 gene encodes a 46 kDa protein that inhibits nuclear localization and suppresses transcriptional activity. It is the main structural element of extracellular matrices in connective tissue and can be found in all organisms. It was also found to be enriched in the protein digestion and absorption and platelet activation processes, and to be related to extracellular matrix remodeling. Few reports have indicated its role in pathological process in multiple cancers, the immune system and angiogenesis (Meng et al., 2019; Watanabe et al., 2016). COL5A2 induces unrestrained cell cycle progression, and cell proliferation, accompanied by upregulation of VEGF and P53 expression (Zeng et al., 2018; Park et al., 2017). In addition to promoting cell proliferation, COL5A2 also promotes tumor progression. via disrupted coagulation, hypoxia and UV exposure since it plays an important role in regulating both programmed cell death and cell proliferation (Ren et al., 2021). CircANRIL was also found to be associated with cell differentiation, proliferation and apoptosis. However, the cellular biological role of circANRIL, COL5A2 and the mechanisms underlying their relationship remains unknown.

The WDR3 gene is a protein-coding gene located on chromosome 1p12-p13 (25) in a region that is related to tumors (Smedley et al., 2000). As a nuclear protein consisting of 10 WD repeat units. WDR3 regulates proteins characterized by the presence of several WD motifs (also known as the Trp-Asp or WD40 motifs). These proteins are involved in cell cycle progression, signal transduction and apoptosis (Dhiani & Mehellou, 2020; McMahon et al., 2010; Liu et al., 2011). UTP12 is the homolog of WDR3 in yeast, and it is a part of the pre-rRNA processing complex that is essential for rRNA processing and synthesis of the small ribosomal subunit (Dragon et al., 2002). There are human WDR3 orthologs, ribosomal 18S RNA processing by the IGF-I-responsive WDR3 protein was found to be related to p53 function in cancer cell proliferation (McMahon et al., 2010).

Notably, COL5A2 and WDR3 are related to cell proliferation, as previously reported. To the best of our knowledge, no previous studies have investigated the affects of these two genes on circANRIL or ANRIL expression, However, their known mechanisms in regulating cell proliferation and signaling pathways in other diseases may indirectly explain their effects on circANRIL.

The limitation of our study is that we only assessed one dataset from circANRIL-OE cells. Although our results cannot be directly applied to humans, they will be of help to our further research. Based on our analysis, we cannot infer the potential mechanism of the hub genes since molecular biological studies are needed to verify the potential mechanism *in vivo* and *in vitro*. Encouragingly, the development of laboratory techniques will provide new methods for determining the biological features and roles of circANRIL in the pathophysiological of processes of multiple diseases.

## Conclusions

In conclusion, 179 up-regulated DEGs, one KEGG pathway, and two hub genes and their signaling pathways were identified in this research. Only COL5A2 and WDR3 were identified to circANRIL function, warranting further studies to explore their application in the clinical setting. However, molecular biological experiments are required to confirm the function of the identified genes in regulating circANRIL expression.

## Supplemental Information

10.7717/peerj.13135/supp-1 Supplemental Information 1 ANRIL and circANRIL expression in different groups

10.7717/peerj.13135/supp-2 Supplemental Information 2 Number of differentially expressed genes among different groups

10.7717/peerj.13135/supp-3 Supplemental Information 3 Enriched GO terms and KEGG pathways of the identified DEGs

10.7717/peerj.13135/supp-4 Supplemental Information 4 Topological properties analysis of PPI network and the selected nodes

10.7717/peerj.13135/supp-5 Supplemental Information 5 The ceRNA network cirRNA-miRNA pairs extracted by Targetscan

10.7717/peerj.13135/supp-6 Supplemental Information 6 miRNA-target gene pairs miRNA-target gene pairs predicted by miRwalk 2.0 et al.

10.7717/peerj.13135/supp-7 Supplemental Information 7 ceRNA network and main circANRIL-miRNA-mRNA pairs ceRNA network constructed by cytoscape

10.7717/peerj.13135/supp-8 Supplemental Information 8 mRNA expression levels of the 6 hub genes verified by qRT-PCR analysis

## Additional Information and Declarations

### Data Availability

The following information was supplied regarding data availability: The data is available at NCBI SRA: PRJNA756043. The other raw data are available as Supplementary Files.
